# Factors governing the adoption of artificial intelligence in healthcare providers

**DOI:** 10.1007/s44250-022-00004-8

**Published:** 2022-10-31

**Authors:** Thomas H. Davenport, John P. Glaser

**Affiliations:** 1 Operations and Information Management Division, Babson College, Wellesley, MA 02457 USA; 2 Harvard Medical School, Boston, MA 02115 USA

## Abstract

Artificial intelligence applications are prevalent in the research lab and in startups, but relatively few have found their way into healthcare provider organizations. Adoption of AI innovations in consumer and business domains is typically much faster. While such delays are frustrating to those who believe in the potential of AI to transform healthcare, they are largely inherent in the structure and function of provider organizations. This article reviews the factors that govern adoption and explains why adoption has taken place at a slow pace. Research sources for the article include interviews with provider executives, healthcare IT professors and consultants, and AI vendor executives. The article considers differential speed of adoption in clinical vs. administrative applications, regulatory approval issues, reimbursement and return on investments in healthcare AI, data sources and integration with electronic health record systems, the need for clinical education, issues involving fit with clinical workflows, and ethical considerations. It concludes with a discussion of how provider organizations can successfully plan for organizational deployment.

The potential of artificial intelligence to transform every aspect of medicine and healthcare is real. It’s vital for healthcare industry leaders who are embarking on this AI journey to understand and maximize its benefits. However, it is difficult to understand the potential maturity of a technology when there is both substantial hype and skepticism about the application of AI to human health. This difficulty is compounded because AI is not a single technology but several, encompassing diverse capabilities and applications.

While there are rapidly–growing numbers of AI innovations in healthcare research labs, relatively few have yet been fully deployed in provider organizations. Healthcare is different from most industries in the extent to which it must rely on public scientific methods to introduce new products and practices. There is a significant regulatory machine that exists, e.g., at the FDA, to ensure that scientific rigor is followed. Most patients appreciate the conservative approach to new treatments. Studies to determine the clinical utility of incorporating AI into clinical practice will take years: to conduct each study, to publish the results, for the medical community to accept the results and alter clinical practice, and for payers to approve reimbursement.

The development and introduction of most consumer-oriented AI products and services, such as driving assistance and autonomy, do not undergo this degree of public scientific rigor. Therefore, adoption of AI in healthcare has been slower than in several other industries, although some types of AI use cases are further along in the adoption process than others. Healthcare providers face the issues of how to accelerate the deployment of AI and overcome barriers to adoption. In this article we describe the key factors that govern AI adoption in provider organizations (primarily hospitals and healthcare systems), and discuss how provider executives can speed adoption processes if desired.

## Clinical vs. administrative applications

While clinical applications of AI are perhaps more exciting, administrative applications—improving payment processes, limiting fraud, or scheduling operating rooms more efficiently—are likely to be much easier to implement. Better and less expensive healthcare administration through AI is currently in reach [1], and provider organizations should seriously consider investing in AI for this purpose. The primary objective of these use cases is reducing administrative costs. While this goal is most desirable in the U.S., which spends more per capita on healthcare than any other country and spends 34% of those costs on administration [2], there is room for administrative cost reductions almost everywhere.

Administrative processes for AI adoption aren’t subject to regulatory approval. The consequences of errors resulting from AI–based decisions are much less problematic in administrative applications than those that impact a patient. When the government is the payer, relevant administrative applications have to comply with its prescribed reimbursement processes, but for internal administration, providers are free to employ AI in any way that benefits them. In addition, the economic return from administrative AI is more under the control of the health system than with clinical applications, which generally require that payers and regulators are also involved.

Many provider institutions—particularly in the U.S. but in other countries as well—are already applying AI for administrative purposes. They work directly with payers, for example, to smooth and speed claims or prior authorization processes. They look for ways to identify patients who need help paying their medical bills—sometimes even in advance of incurring them. They use AI to ensure proper disease coding on bills, or to make appointment scheduling easier for patients.

What is typically required for administrative AI applications to be deployed is similar to administrative AI in other industries. The application has to be effective, leading to better decisions or higher productivity. It must be integrated with existing systems and processes, which may be easier if the AI application is procured from an existing vendor. There may also be training and upskilling necessary for those who will use the AI system.

## Regulatory approval

AI for clinical purposes—specifically, diagnosis, treatment, and monitoring—is eventually going to impact every healthcare organization in one or more of these categories as vendors incorporate these capabilities into existing products or develop new ones. Some applications will need regulatory approval depending on the extent to which they are directly involved in patient care. The U.S. Food and Drug Administration classifies certain applications of AI as “software-based medical devices” and has regulated them accordingly through several different pathways. As of mid-2022, the FDA has approved almost 350 such applications [3]. Europe does the same through its CE Mark, and has approved somewhat more devices than the FDA. In both regions, the bulk of approved devices involve analysis of sensor data from patients, or radiological imaging applications. Most are from companies, rather than healthcare provider organizations.

However, regulatory clearance alone can’t guarantee that an AI-based application will always work as billed in clinical use. A 2021 commentary article recommended that clinicians be able to answer the questions below when considering adopting AI [4]. They apply to all specialties, not just radiology, although AI algorithms do often perform differently across different imaging devices. The questions are:

- (1) What is the scope of products that are available for my intended use?
- (2) How were the models trained and how were they validated?
- (3) Once purchased, will an AI application perform as expected in my practice? How can I monitor the performance of the model after deployment?

## Reimbursement and return on investment

Healthcare providers around the world must worry about how to pay for any innovation in healthcare, including AI. In the best case, innovations pay for themselves, allowing providers to offer better care at the same cost, or to offer the same quality care at lower cost. Some AI-based innovations may fit this best-case scenario, but many will require payer approval and reimbursement for providers to afford to adopt them. In the UK, the National Health Service announced in 2019 that it would begin to reimburse for AI-based care in 2020 to incent more rapid adoption, though details have been sketchy [5]. The NHS is also investing about £250,000 in AI for healthcare through the NHS AI Lab [6].

In China, in part because of the COVID-19 pandemic, the Chinese National Health Commission approved reimbursement for online consultations using AI and other digital tools in 2020. China has seen massive growth in the use of AI for general practitioner advice, which can determine whether a face-to-face consultation is required. We could find no evidence that some of the more advanced image detection use cases are reimbursed (or in clinical practice) in China, although there are plenty of startups in that space.

At this writing fewer than ten AI-based applications—including one for diagnosing blood clots in the brain and another for diabetic retinopathy—have been approved for reimbursement by the U.S. Centers for Medicare and Medicaid Services (CMS), which pays for about half of U.S. healthcare [7]. It is expected that private insurers will follow the lead of CMS, but they haven’t done so publicly yet.

As healthcare moves to value-based payment models, which require providers to support their patients’ *health* rather than simply providing health *care* for particular illnesses or medical issues, reimbursement for AI-based innovations may become more common. However, the movement to value-based care is very slow. The COVID-19 pandemic has made it even slower as providers focus on very short-term measures to rein in costs and meet immediate patient needs. Patient volume in most health systems has also not recovered to pre-pandemic levels. When value-based care does become a reality, provider organizations will need to understand and manage their patient populations in new ways, and AI-enabled decisions may be their best route to doing so.

Today, however, many provider-based clinical uses of AI are experimental. They are neither approved by the FDA or other regulatory body, nor approved for reimbursement by payers. Few generate a high level of productivity improvement. Therefore, they provide little return on investment. As a result, the provider organizations that currently support extensive AI development are likely to be large, research-focused, and relatively wealthy.

## Data and EHR integration

Data is the fuel of AI, and is required to train machine learning models. Despite some progress over the past couple of decades, healthcare data is generally still as fragmented and siloed as the healthcare system that creates it, at least in the U.S. Most hospitals and group medical practices have their own EHR data and little else. Unless they are also providers, payers generally have only claims data, although some are partnering with providers to get access to their EHR data. It is extremely rare to have all a patient's healthcare data—across all providers and payers—available in one easily–accessible repository. That means that data used to train machine learning models will of necessity be limited and will probably not encompass all of a patient’s interactions with the healthcare system. Even within a particular institution, data scientists or engineers will often need to spend considerable time integrating and curating data.

Some national healthcare systems have a common EHR system, which makes it relatively straightforward to both gather data to train models and to integrate new AI-based scoring systems into clinical practice. For example, the U.K.’s NHS, which doesn’t have an overall common EHR system but does have one for general practitioners, has created and deployed an “Electronic Frailty Index” from EHR data. The machine learning model creates a score for elderly patients that is integral within the EHR system. If the GP sees a patient with a severe or moderate frailty index, special care measures (such as a medication review or falls risk assessment) are mandated or recommended [8].

Limited data integration does not impact all clinical AI algorithms. AI methods directed to interpreting radiology images, for example, do not require the integration of a broad range of EHR data. However, exciting AI opportunities, such as comparative effectiveness determination and understanding the factors that increase the risk of disease, will be hobbled by poor interoperability. Moreover, as the range of health-related data increases to include, for example, social determinants of health and wearable sensors, limited data integration will become increasingly problematic. The potential value of AI may drive better data standards, integration and sharing over time.

## Clinician education and workflow

Clinicians will need substantial education in AI to use it effectively in clinical practice. Medical schools have yet to integrate AI across the curriculum (and only a few have addressed digital or information technologies of any type, or personalized/precision medical care, in formal courses). Although it’s early days for this type of training, some courses are beginning to be offered, particularly in a few AI-oriented specialty areas. For example, the Radiological Society of North America has announced an imaging AI certificate that radiologists can earn online.

However, such programs are still relatively rare. They are largely absent in other fields where AI is increasingly capable of image analysis, such as pathology [9] and ophthalmology [10]. Professional associations in these specialties need to move quickly to improve specialists’ awareness and skills.

Clinicians may resist using AI systems that don’t fit well into clinical workflows. This complaint has been leveled against EHRs in general, but they are so critical to modern medical practice that most physicians use them anyway. If AI systems require separate systems, apps, APIs, or logins, they are much less likely to be adopted. Since EHRs have become the dominant technology that structures clinical workflows, AI will need to be integrated into those systems to be widely deployed with any success.

Some specialties involve tasks and workflows that are more conducive to AI use than others. A McKinsey study, for example, classified “automatable hours” in clinical roles in an analysis of the impact of AI on healthcare jobs in Europe [11]. It found that “family and general practitioners” have the highest percentage (but only 12%) of automatable hours among physicians. Physical positioning of patients is an example of an “unpredictable physical” task—a factor that diminishes the likelihood of automation in the McKinsey study. Chiropractors have the lowest percentage of automatable work hours—2%—among all clinicians.

Empathy and understanding of mental health (“interfacing with stakeholder” tasks) were deemed unlikely with AI in the AI impact study; psychiatrists had the fewest automatable hours among physicians. However, for some conditions (anxiety, depression, and substance use) intelligent, emotion-oriented chatbots that employ cognitive behavior therapy may be able to help patients, particularly since there is a serious shortage of mental health professionals in many countries [12].

Specialists such as radiologists and pathologists who do not normally see patients in person may be more affected by AI. Image interpretation is a substantial component of their jobs, and they often communicate with patients and other physicians through reports that could be automatically generated. However, these specialists do perform a number of tasks that are not likely to be automated soon [13].

Clinical professionals whose primary focus is caring for patients across a broad spectrum of needs, such as nurses, seem unlikely to be greatly affected by AI. Those who primarily provide diagnosis and advice, such as physicians, seem more likely to be affected. Perhaps the greatest impact from AI and related automation capabilities will involve administrative workers in healthcare rather than clinical ones.

## Ethical considerations

Ethical AI is a concern for all industries but a greater one for healthcare. The ethical principles developed by the World Health Organization in 2021 for AI use in healthcare address such issues as protecting human autonomy; ensuring transparency, explainability, and intelligibility; and fostering responsibility and accountability. Complying with such principles, however reasonable they seem, will not be easy or even possible for many AI systems. Most deep learning models for radiological image analysis, for example, are today neither transparent nor explainable.

Leading provider organizations in the adoption of AI have begun to specify their ethical principles, but few have created a management structure to ensure that all AI applications—those developed in-house as well as those from vendors—comply with the principles. We expect that close adherence to ethical considerations will slow down the development and adoption of clinical AI applications.

## Planning for AI adoption

The advantage of a deliberate pace for AI adoption is that It gives healthcare organizations time to plan and adapt. Positioning a provider organization for success depends on several factors.

Certainly AI adoption will move faster in organizations that declare adoption to be a strategic priority than in organizations that view it as a novelty or niche technology. Some research and innovation-focused providers, such as Mayo Clinic for example, have many AI projects underway and have created new organizational roles and structures to facilitate the growth of AI across the organization [14].

In terms of other attributes, organizations that have deployed core transaction applications, such as electronic health records and revenue cycle applications, will be better positioned to incorporate AI into the workflow. This transformation will also be simpler and faster in organizations that have a base of applications from one vendor across the enterprise than those with applications from multiple vendors. Provider organizations are likely to adopt AI more quickly and smoothly if they already know how to move new technologies from pilot to broad deployment and manage the accompanying workflow and/or cultural changes.

AI technologies are changing rapidly, but healthcare processes and professionals change much more slowly. The value of the technology, however, is sufficiently great that all substantial healthcare providers should begin to evaluate AI technologies and consider how they might help to transform clinical and administrative processes.

## Data Availability

A review article, so no data.
